# Papaya (*Carica papaya* L.) Peel as a Sustainable Source of Vision-Related Carotenoids Through Green Extraction Optimization

**DOI:** 10.3390/molecules31132253

**Published:** 2026-06-26

**Authors:** Noreima Barroso-Torres, M. Gloria Lobo, Eva Dorta

**Affiliations:** 1 Instituto Canario de Investigaciones Agrarias (ICIA), Valle de Guerra, 38270 San Cristóbal de la Laguna, Spain; globo@icia.es (M.G.L.); edorta@icia.es (E.D.); 2 Escuela de Doctorado y Estudio de Posgrado, Universidad de La Laguna, 38200 San Cristóbal de la Laguna, Spain

**Keywords:** antioxidant activity, response surface methodology (RSM), by-products, health, bioactive

## Abstract

Agro-industrial by-products represent a sustainable and underutilized source of bioactive compounds with potential applications in human health. Among them, papaya (*Carica papaya* L.) peel, typically discarded during industrial processing, constitutes a promising and underexploited matrix for carotenoid recovery and valorization. In this study, different extraction strategies were evaluated and compared to identify the most efficient approach for carotenoid recovery. Total carotenoid contents of 7.13 ± 0.32, 5.35 ± 0.24, and 4.44 ± 0.38 µg β-carotene/100 g dry weight (DW) were obtained, respectively. The conventional extraction was further optimized using response surface methodology (RSM) to maximize carotenoid recovery and antioxidant activity. The extracts obtained under the optimized conditions were characterized by spectrophotometric analysis, HPLC-DAD, and in vitro antioxidant assays (DPPH and ORAC-FL), exhibiting antioxidant capacities of 528.4 ± 43.3 µmol TE/100 g DW and 5.0 ± 0.5 mmol TE/100 g DW, respectively. The carotenoid profile revealed lutein as the predominant compound (1414.28 µg/100 g fresh weight (FW)), followed by violaxanthin (629.55 µg/100 g FW), zeaxanthin (624.58 µg/100 g FW), β-cryptoxanthin (531.23 µg/100 g FW), and β-carotene (278.82 µg/100 g FW), while lycopene was not detected. The predominance of xanthophylls, particularly lutein, highlights the potential of papaya peel as a source of carotenoids that have been associated with visual health in previous studies, supported by its significant antioxidant activity. Overall, papaya peel is confirmed as a valuable and sustainable source of carotenoids, particularly xanthophylls associated with visual health, supporting its potential use in the development of functional ingredients. These findings contribute to circular economy strategies and support the sustainable production of bioactive compounds with potential applications in functional food and nutraceutical formulations.

## 1. Introduction

Carotenoids are bioactive compounds that play essential roles in human health due to their antioxidant capacity and diverse biological functions, including serving as provitamin A precursors, modulating immune responses, and contributing to visual and macular health. Their deficiency has been associated with an increased risk of inflammatory diseases (chronic obstructive pulmonary disease, measles-related infections, inflammatory bowel disease…) and conditions associated with oxidative stress [1]. Particularly relevant are disorders associated with vitamin A deficiency, including impaired immune response and visual disorders, such as xerophthalmia and night blindness [2]. In addition, specific carotenoids, such as β-carotene and β-cryptoxanthin, can be converted into retinol in the human body, serving as provitamin A compounds. In contrast, lutein and zeaxanthin are especially relevant for ocular health because they accumulate in the macular region of the retina, where they help protect against age-related macular degeneration [3]. Likewise, violaxanthin, an epoxy xanthophyll naturally present in several fruits and vegetables, has gained increasing attention due to its antioxidant activity and its potential role in protecting retinal tissues against oxidative and light-induced damage.

Collectively, these compounds have attracted considerable attention due to their beneficial effects on visual health, particularly their role in protecting retinal tissues against oxidative stress and age-related ocular disorders. Macular pigments are mainly composed of the xanthophyll carotenoids lutein, zeaxanthin, and meso-zeaxanthin, which are responsible for the characteristic yellow coloration of the macula lutea. While lutein and zeaxanthin must be obtained through the diet, meso-zeaxanthin can be partially formed from lutein metabolism in the retina [4]. Therefore, the regular consumption of carotenoid-rich foods is considered essential to maintain macular pigment density and visual function. These properties have motivated the incorporation of carotenoid-rich ingredients into functional foods and nutraceutical formulations intended to support visual health [5].

The relevance of dietary carotenoids in ocular health has been further supported by clinical studies such as the Age-Related Eye Disease Study 2 (AREDS2), in which supplementation with lutein and zeaxanthin demonstrated protective effects against the progression of age-related macular degeneration (AMD) [6]. These findings reinforce the relevance of carotenoid-rich foods and ingredients as dietary sources of compounds associated with retinal function and protection against oxidative stress.

Fruit and vegetable matrices are major dietary sources of carotenoids with established roles in ocular health, where they exert antioxidant protection against oxidative damage in both the retina and the lens. Epidemiological evidence consistently associates higher dietary carotenoid intake with a reduced risk of age-related macular degeneration and cataract formation. Importantly, the biological efficacy of carotenoids is strongly influenced by the food matrix, which enhances their bioaccessibility and absorption compared with isolated compounds, supporting whole-food consumption as a more effective strategy for visual health. Within this context, papaya (*Carica papaya* L.) is a relevant source of provitamin A carotenoids and xanthophylls involved in retinal protection and visual function. Papaya is a tropical fruit cultivated in many regions of the world, with the Canary Islands (Spain) the main producer in Europe, where it represents a crop of increasing economic and nutritional relevance. Its industrial processing generates large amounts of by-products, mainly peel and seeds, that are commonly discarded despite their richness in bioactive molecules. In addition, during packing operations, fruits that fail to meet commercial quality standards because of defects in appearance, size, or physical damage are discarded during postharvest grading and sorting processes, generating substantial amounts of by-products.

The valorization of these by-products offers opportunities to strengthen sustainability and circular economy strategies in the agri-food sector, in line with current European policies promoting resource efficiency and waste reduction [7].

Importantly, recovering carotenoid-rich fractions from these by-products offers an opportunity to simultaneously address sustainability challenges and generate value-added ingredients containing compounds associated with visual health in previous studies.

Efficient recovery of carotenoids from plant matrices is challenging because of their sensitivity to light, oxygen, and heat, as well as the complexity of fruit tissues. Traditional solvent-based methods are widely used but often involve toxic, non-sustainable solvents. In recent years, there has been increasing attention toward green extraction technologies, such as ultrasound-assisted extraction (UAE) and microwave-assisted extraction (MAE), which aim to improve yield, reduce solvent consumption, and minimize environmental impact [8]. Optimizing such methodologies is essential to ensure high carotenoid recovery while maintaining their stability and bioactivity.

Moreover, the valorization of fruit by-products, such as papaya peel, as raw materials for carotenoid recovery represents a sustainable alternative that aligns with bioeconomy and circular economy principles [7]. Using these residues not only reduces environmental burdens but also provides a cost-effective source of bioactive compounds, reinforcing the relevance of developing optimized and efficient extraction methodologies for their recovery and potential application in health-oriented industries.

Although response surface methodology (RSM) has increasingly been applied to optimize carotenoid extraction from plant materials and agro-industrial by-products, studies specifically focused on papaya peel remain limited. Furthermore, comparative evaluations of conventional and assisted extraction technologies under a common experimental framework are still scarce [9,10]. Beyond extraction optimization itself, the novelty of the present study lies in the targeted valorization of papaya peel as a sustainable source of carotenoids associated with visual health. In addition to comparing extraction technologies, the work integrates carotenoid recovery, antioxidant evaluation, and detailed HPLC-DAD characterization of compounds of nutritional interest, particularly lutein and zeaxanthin, within a circular economy framework. Therefore, the study contributes to the development of value-added functional ingredients from agro-industrial by-products and expands the potential applications of papaya peel beyond conventional waste management strategies.

While several carotenoids identified in papaya peel have been associated with visual health in previous studies, the present work does not aim to evaluate biological effects or ocular outcomes. Instead, it focuses on the optimization of extraction procedures and the characterization of carotenoid-rich extracts obtained from this agro-industrial by-product. In this context, the present study aimed to (i) optimize a vortex-assisted extraction (VAE) method for carotenoids from papaya peel, (ii) compare its performance with ultrasound-assisted (UAE) and microwave-assisted extraction (MAE) techniques, and (iii) evaluate the carotenoid composition and antioxidant capacity of the resulting extracts, with particular emphasis on the identification and quantification of carotenoids associated with visual health according to previous studies, as well as their potential applicability as provitamin A sources.

## 2. Results and Discussion

### 2.1. Preliminary Solvent Screening

Preliminary experiments were conducted to evaluate the effect of different solvent proportions of methanol, petroleum ether, and diethyl ether (1:1:1, 1:2:1, 1:2:2, 1:1:2, and 2:1:1, *v*/*v*/*v*), corresponding to solvent systems commonly employed for carotenoid extraction [11]. Total carotenoid contents determined spectrophotometrically at 450 nm ranged from 5.1 to 6.8 µg β-carotene/g DW for all tested mixtures. No statistically significant differences were observed among the solvent systems (ANOVA, *p* > 0.05), indicating that variations in solvent proportions did not markedly influence carotenoid recovery under the evaluated conditions.

Considering the comparable extraction efficiencies obtained, the 1:1:1 methanol: petroleum ether: diethyl ether mixture was selected for subsequent experiments due to its reproducibility and operational convenience, ensuring robust and consistent extraction performance during the optimization stage.

### 2.2. Ascorbic Acid Determination

The ascorbic acid content of the extracts was determined to evaluate its potential contribution to the measured antioxidant capacity. The obtained concentration was 0.092 ± 0.008 mg ascorbic acid/100 g DW, indicating a very low presence of this compound in the extracts. These results suggest that ascorbic acid did not significantly contribute to the antioxidant activity measured by DPPH and ORAC-FL assays.

Furthermore, the low recovery of ascorbic acid confirms that the extraction methodology employed, based on predominantly non-polar solvent systems, was not suitable for the efficient extraction of this highly polar compound. Therefore, the antioxidant capacity observed in the extracts can be mainly attributed to lipophilic bioactive compounds, particularly carotenoids.

### 2.3. Comparison with Assisted Extraction Methods

The extraction performance of the conventional vortex-assisted method (VAE), ultrasound-assisted extraction (UAE), and microwave-assisted extraction (MAE) was evaluated using a 1:1:1 methanol:petroleum ether:diethyl ether solvent mixture. Total carotenoid contents, expressed as µg β-carotene per 100 g dry weight (µg β-carotene/100 g DW), were 7.13 ± 0.32 for VAE, 5.35 ± 0.24 for UAE, and 4.44 ± 0.38 for MAE. Statistical analysis revealed significant differences among the extraction techniques (*p* < 0.05), with the VAE showing significantly higher carotenoid recovery than both assisted extraction methods (UAE and MAE).

The lower extraction efficiency observed for UAE and MAE may be related to the physicochemical characteristics of carotenoids and the nature of the papaya peel matrix. Carotenoids are known to be susceptible to oxidation, isomerization, and thermal degradation. In the case of MAE, localized heating effects may have promoted partial degradation of these compounds, reducing their recovery. Similarly, although the UAE enhances mass transfer through cavitation phenomena, the formation of reactive species and localized high-energy conditions may contribute to the degradation of oxidation-sensitive molecules such as carotenoids. Furthermore, the papaya peel used in this study was freeze-dried and finely ground before extraction, which likely facilitated solvent penetration and carotenoid release. Under these conditions, the conventional vortex-assisted extraction appeared sufficient to achieve efficient recovery, limiting the additional benefits typically associated with assisted extraction technologies.

Based on these results, the VAE was selected for subsequent optimization experiments, as it provided the highest extraction efficiency under the evaluated conditions. From a practical perspective, VAE is a simpler and less energy-intensive approach, avoiding specialized equipment such as ultrasonic generators or microwave reactors, which require specific optimization and operational expertise. In addition, its ease of operation and scalability make it more feasible for industrial applications. Therefore, considering its comparable performance and greater operational simplicity, the conventional method was selected for further optimization by response surface methodology (RSM).

### 2.4. Optimization of the Conventional Extraction Method

The VAE protocol was optimized using response surface methodology (RSM). A three-factor design was applied to evaluate the influence of (i) homogenization time (1.0–2.0 min), (ii) solid-to-solvent ratio (0.25–0.40 *w*/*v*), and (iii) number of extraction cycles (1–3) on carotenoid yield and antioxidant activity. The RSM models exhibited good predictive performance, with close agreement between predicted and experimental values (Table 1), as reflected by the high coefficient of determination (R^2^ = 0.954) and adjusted coefficient of determination (adjusted R^2^ = 0.852), indicating the adequacy of the model to describe the extraction process. The relationship between the evaluated extraction variables and total carotenoid content was described by the following second-order polynomial model: TCC = 54.8165 + 251.615A + 29.800B − 50.0658C − 421.796A^2^ + 95.500AB + 48.150AC − 36.1617B^2^ + 7.385BC + 8.79458C^2^, where A corresponds to the solid-to-solvent ratio, B to vortex homogenization time, and C to the number of extraction cycles.

The results showed that carotenoid recovery was strongly influenced by the extraction conditions. In general, higher solid-to-solvent ratios (0.40 *w*/*v*) and an increased number of extraction cycles (*n* = 3) led to improved carotenoid yields, highlighting the importance of sufficient solvent availability and repeated extraction steps to enhance the solubilization and diffusion of these hydrophobic compounds from the plant matrix. In contrast, lower solvent ratios (0.10–0.25 *w*/*v*) and fewer extraction cycles resulted in significantly reduced carotenoid recovery, suggesting incomplete extraction under these conditions.

Regarding homogenization time, intermediate values (around 1.0–1.5 min) were found to be more favorable, whereas longer times did not result in substantial improvements and, in some cases, were associated with lower carotenoid yields. This behavior may be related to the known susceptibility of carotenoids to oxidation and degradation during processing, although no direct degradation measurements were performed in the present study. Alternatively, the results may indicate the existence of an optimal balance between extraction efficiency and compound stability under the evaluated conditions.

These trends are consistent with the highest experimental value observed (18.02 ± 0.66 mg β-carotene/100 g DW), obtained under conditions combining a high solvent ratio and appropriate extraction parameters, supporting the relevance of optimizing these variables. However, although experiment 13 yielded the highest carotenoid concentration, a greater deviation between the experimental and model-predicted values was observed under these conditions. In contrast, experiment 15 showed a closer agreement with the values predicted by the RSM model, indicating better model reliability and robustness for process optimization.

**Figure 1:**
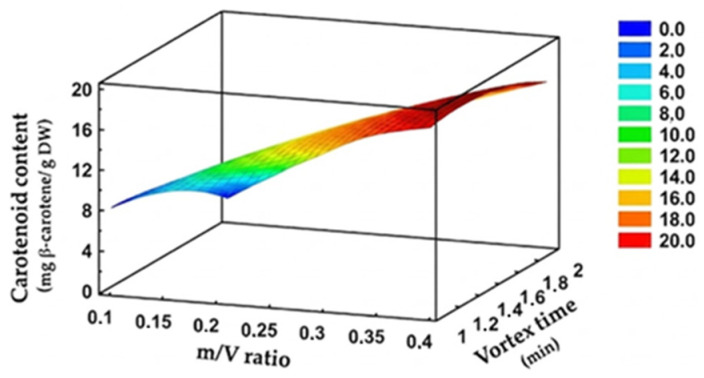
Response surface plot showing the effect of vortex time (min) and solvent-to-sample ratio (m/V) on total carotenoid content (mg β-carotene/g DW) in papaya peel extracts. The number of extraction cycles was fixed at three.

Overall, the interaction between extraction cycles, solvent ratio, and homogenization time played a key role in maximizing carotenoid recovery. Based on the model predictions and the consistency between predicted and experimental responses, the optimal extraction conditions were established as a solid-to-solvent ratio of 0.40 (*w*/*v*), 3 extraction cycles, and 1.25 min of vortex homogenization. Response surface plots illustrating factor effects are presented in Figure 1.

### 2.5. Antioxidant Capacity

**Figure 2:**
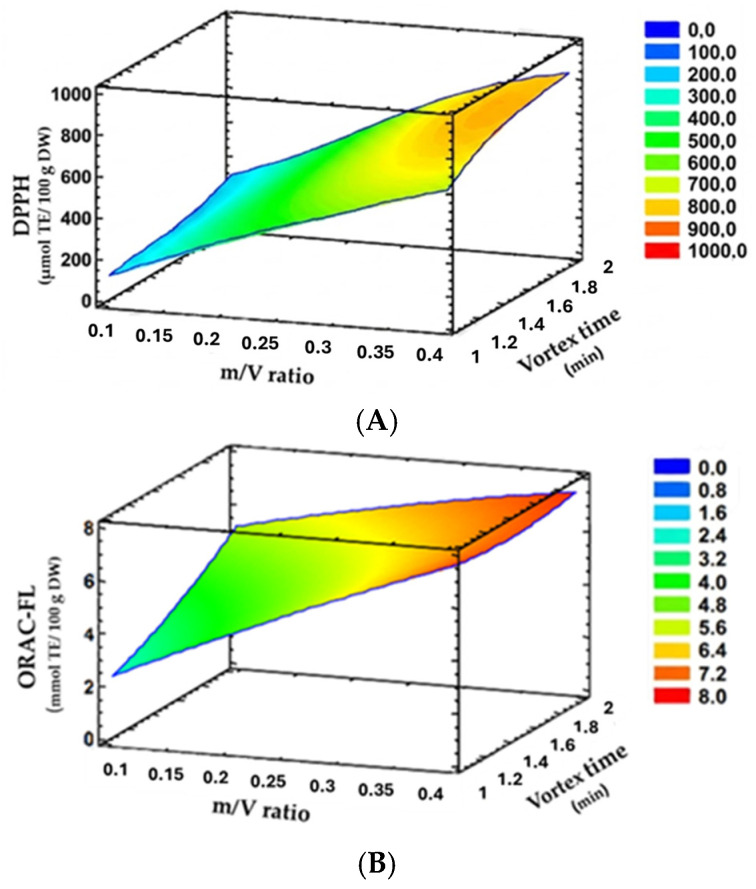
Response surface plots showing the effect of vortex time (min) and solvent-to-sample ratio (m/V) on antioxidant capacity in papaya peel extracts: (**A**) DPPH analysis (µmol TE/100 g DW) and (**B**) ORAC-FL analysis (mmol TE/100 g DW). The number of extraction cycles was fixed at three.

In addition to carotenoid yield, the influence of extraction variables on antioxidant activity was evaluated using DPPH and ORAC-FL assays (Table 2, Figure 2). The RSM models showed good predictive performance for both responses, with satisfactory agreement between experimental and predicted values (R^2^ = 0.867 for DPPH and 0.84 for ORAC-FL, R^2^ = 0.845 and adjusted R^2^ = 0.567 for ORAC-FL), confirming the adequacy of the models. The relationship between the extraction variables and antioxidant activity was described by the fitted second-order polynomial models. For DPPH, the response was expressed as follows: DPPH = 990.404 + 1020.184A − 776.048B − 220.440C + 99.630A^2^ − 237.733AB + 374.733AC + 202.887B^2^ + 71.380BC + 18.802C^2^, whereas the ORAC-FL response was described by the following: ORAC = 3.53724 + 25.4486A − 4.6375B − 0.00745833C − 7.12222A^2^ − 9.490AB + 1.58333AC + 1.034B^2^ + 1.8065BC − 0.55475C^2^, where A corresponds to the solid-to-solvent ratio, B to vortex homogenization time, and C to the number of extraction cycles.

The results revealed that antioxidant activity followed trends similar to those observed for carotenoid content, indicating a strong relationship between carotenoid recovery and radical scavenging capacity. In general, higher solid-to-solvent ratios (0.40 *w*/*v*) and an increased number of extraction cycles (*n* = 3) resulted in enhanced antioxidant activity, with the highest values reaching 906.4 ± 48.3 µmol TE/100 g DW for DPPH and 7.7 ± 0.8 mmol TE/100 g DW for ORAC-FL. These findings suggest that improved extraction efficiency leads to a higher recovery of bioactive compounds responsible for antioxidant activity.

Regarding homogenization time, intermediate values again proved to be more favorable, whereas prolonged extraction times did not significantly improve antioxidant capacity and, in some cases, resulted in slightly lower values. This behavior may be attributed to the potential degradation or oxidation of sensitive compounds, including carotenoids, under extended mechanical treatment.

Both methods confirmed strong radical scavenging activity and oxygen radical absorbance capacity (Table 2). These results are consistent with the high carotenoid content of the extracts and are in line with values previously reported for papaya peel [8]. Notably, the observed antioxidant capacity, together with the carotenoid profile identified, highlights the potential value of papaya peel as a source of bioactive carotenoids, including compounds previously associated with visual health in the scientific literature.

Comparison with previously reported data provides further insight into the antioxidant potential of papaya peel. The results obtained in this study showed antioxidant capacities of 528.4 ± 43.3 mmol TE/100 g DW (DPPH) and 5.0 ± 0.5 mmol TE/100 g DW (ORAC-FL), which fall within the range reported for papaya-derived materials and other tropical fruit by-products. For instance, a DPPH radical scavenging activity equivalent to 327.18 mmol TE/100 g DW has been previously reported for papaya peel [12], indicating values of the same order of magnitude as those obtained in the present study. These differences may be attributed to variations in cultivar, extraction methodology, maturity stage, and processing conditions [13]. Similarly, ORAC values ranging from 1.2 to 2.6 mmol TE/100 g DW have been reported for papaya-derived materials processed under different conditions, including thermal drying and fermentation, both of which are known to affect carotenoid stability and antioxidant activity [14].

Overall, the antioxidant capacity observed in this study is consistent with the presence of bioactive compounds in papaya peel, particularly carotenoids. Importantly, these findings highlight papaya peel as a promising source of carotenoids, particularly lutein and zeaxanthin, which have been associated with visual health in previous studies. Nevertheless, the present work was limited to extraction optimization, carotenoid characterization, and antioxidant assessment; therefore, no conclusions can be drawn regarding biological effects on ocular tissues or visual function [15,16].

To further evaluate the relationship between carotenoid content and antioxidant activity, a Pearson correlation analysis was performed using the experimental values obtained under all extraction conditions. Total carotenoid content showed a strong positive correlation with both DPPH (r = 0.897, *p* < 0.001) and ORAC-FL (r = 0.822, *p* < 0.001). These results support the contribution of carotenoids to the antioxidant properties of papaya peel extracts and reinforce the observed association between carotenoid recovery and antioxidant capacity.

The predictive capacity of the optimized RSM model was further validated by comparing the extraction performance under the optimal conditions with a randomly selected extraction condition (Table 3). The optimized parameters (3 extraction cycles, 0.40 *w*/*v* solid-to-solvent ratio, and 1.25 min vortex homogenization) resulted in substantially higher carotenoid recovery and antioxidant activity than the non-optimized condition. Total carotenoid content increased from 6.76 ± 0.46 to 13.54 ± 0.71 mg β-carotene/100 g DW, while DPPH and ORAC-FL values also showed notable improvements under optimized conditions. These results confirm the suitability and robustness of the RSM approach for maximizing carotenoid extraction efficiency and antioxidant potential from papaya peel.

### 2.6. Analysis of Carotenoid Profiles by HPLC

**Figure 3:**
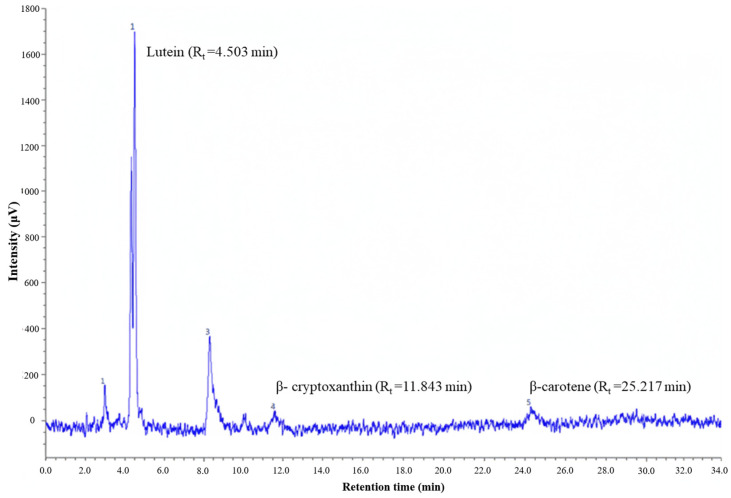
HPLC-DAD chromatogram of carotenoids extracted from papaya (*Carica papaya* L.) peel under optimized conditions. Detection was carried out at 450 nm. The main peaks correspond to target carotenoids associated with visual and macular health, identified according to their retention times and spectral characteristics. Peak assignments are as follows: (1) lutein, Rt = 4.503 min; (4) β-cryptoxanthin, Rt = 11.843 min; (5) β-carotene, Rt = 25.217 min. Rt: retention time.

Following the development of external calibration curves using commercially available carotenoid standards, the chromatographic profiles of papaya peel extracts obtained under optimized conditions were analyzed by HPLC-DAD (Figure 3). This approach enabled the accurate identification and quantification of the main carotenoids present in the extracts, which are directly associated with ocular health and vision-related disorders, including lutein, zeaxanthin, β-cryptoxanthin, and β-carotene, while lycopene was not detected in the analyzed samples.

Among the identified compounds, lutein was the predominant carotenoid, reaching values of 1414.28 µg/100 g FW. The concentrations of the remaining carotenoids were 629.55 µg/100 g FW for violaxanthin, 624.58 µg/100 g FW for zeaxanthin, 531.23 µg/100 g FW for β-cryptoxanthin, and 278.82 µg/100 g FW for β-carotene. These results highlight the abundance of carotenoids in papaya peel, which are known for their relevance in visual health and antioxidant activity.

In contrast, lycopene was not detected under the analytical conditions employed, suggesting that this carotenoid is either absent or present below the detection limit in papaya peel from mature fruits of the Honey variety. This observation is consistent with previous reports indicating that carotenoid composition in papaya strongly depends on cultivar characteristics, particularly flesh pigmentation and ripening stage, with lycopene being more commonly associated with red-fleshed varieties [11].

Comparison with previously reported data indicates that the lutein content found in this study (1414.28 µg/100 g FW) falls within the same order of magnitude as values described in the literature for similar papaya by-products, such as 922.50 µg/100 g FW reported in a recent study [11]. Similar trends have also been reported for other tropical fruit by-products, which are valuable sources of carotenoids and antioxidant compounds, supporting the growing interest in valorizing these underutilized matrices as functional ingredients. These variations are expected and may be attributed to factors such as cultivar, growing conditions, maturity stage, and extraction methodology, particularly the optimization achieved through the RSM approach applied in this study [17,18].

The carotenoid profile identified in papaya peel is particularly relevant considering that lutein, zeaxanthin, and β-carotene are among the main bioactive compounds currently included in nutritional formulations developed for macular protection and age-related ocular disorders, such as those based on the AREDS2 recommendations [6]. In this context, the presence of these carotenoids in papaya peel reinforces its potential as a dietary source of carotenoids that have been associated with visual health in previous studies.

Although the concentrations detected in the present study are considerably lower than those provided by clinically validated supplementation strategies, making the direct therapeutic use of papaya peel unrealistic, its regular incorporation into carotenoid-rich food formulations could contribute to preventive nutritional approaches aimed at reducing oxidative stress in ocular tissues. This aspect may be particularly relevant in populations with prolonged exposure to artificial blue-light sources from early ages, where adequate dietary intake of macular carotenoids has been associated with retinal protection and visual performance maintenance.

Overall, these findings confirm that papaya peel is a rich source of nutritionally relevant carotenoids, especially lutein and other xanthophylls, reinforcing its potential as a functional ingredient for applications related to eye health and antioxidant protection.

## 3. Materials and Methods

### 3.1. Plant Material

Papaya fruits (*Carica papaya* L., cv. Honey) at commercial maturity (25% orange peel coloration) were harvested from plants cultivated under controlled agronomic conditions at the experimental farm “La Estación” of the Instituto Canario de Investigaciones Agrarias (ICIA), located in Vecindario, Santa Lucía de Tirajana (Gran Canaria, Canary Islands, Spain; 27.844680, −15.430455).

After harvest, the fruits were stored at 20 °C and 85% relative humidity until they reached the consumption stage, defined as 100% orange peel coloration. The fruits were then washed and peeled, and the peels were collected as agro-industrial by-products for subsequent analyses. The peels were immediately frozen at −80 °C, followed by lyophilization (−55 °C, 5–7 days). The freeze-dried material was subsequently ground into a fine powder and stored in airtight containers under dark conditions until further use.

### 3.2. Chemicals and Solvents

All solvents used were of analytical or HPLC grade. Methanol, petroleum ether, acetonitrile, triethylamine, and dimethyl ether were purchased from Scharlau (Barcelona, Spain). Trolox (6-hydroxy-2,5,7,8-tetramethylchroman-2-carboxylic acid), 2,2′-azobis(2-amidinopropane) dihydrochloride (AAPH), fluorescein, and 2,2-diphenyl-1-picrylhydrazyl (DPPH) were obtained from Merck (Darmstadt, Germany). All reagents were used without further purification.

### 3.3. Preliminary Solvent Selection and Chromatography Conditions

A preliminary screening was performed to guide the selection of the extraction solvent. Mixtures of methanol (a polar, water-miscible solvent) and petroleum ether/dimethyl ether (non-polar solvents) were tested in different proportions under identical extraction conditions, consisting of mechanical homogenization using a vortex, followed by centrifugation and recovery of the supernatant. This procedure was repeated for three consecutive extraction cycles. All solvent screening experiments were performed in triplicate. Total carotenoid contents obtained with the different solvent systems were compared by one-way ANOVA followed by Tukey’s test (*p* < 0.05). To prevent carotenoid degradation and isomerization, all extraction procedures were performed under cold conditions and protected from light exposure. This solvent combination, consisting of methanol, diethyl ether, and petroleum ether, was selected based on previous reports, where it has been widely applied for the extraction of carotenoids [11]. Carotenoids, being highly hydrophobic compounds, are more efficiently solubilized in non-polar media, while the use of a polar solvent enhances matrix disruption and penetration [19]. Carotenoid recovery was assessed by (i) spectrophotometric quantification at 450 nm and (ii) chromatographic analysis using HPLC-DAD. Chromatographic analyses were performed on a JASCO LC-4000 (JASCO Corporation, Tokyo, Japan) system equipped with a reversed-phase C30 column (YMC Carotenoid, 250 × 4.6 mm, 5 µm; YMC Co., Ltd., Kyoto, Japan) and a diode array detector (DAD). The separation was carried out at 32 °C using a gradient mobile phase consisting of (A) acetonitrile and (B) methanol:ethyl acetate (50:50, *v*/*v*) containing 0.5% triethylamine. The gradient program started at 80% A and 20% B, increased to 90% A and 10% B at 25 min, and returned to the initial conditions (80% A, 20% B) at 55 min. Before HPLC injection, all extracts were filtered through 0.45 µm membrane filters. The flow rate was set at 1 mL/min, with a total run time of 55 min per injection. The extractant mixture selected from this screening was subsequently used for the optimization of the conventional carotenoid extraction (Section 2.4).

### 3.4. Experimental Design and Optimization

The optimization of carotenoid extraction was carried out using Response Surface Methodology (RSM) based on a Box–Behnken experimental design. Three independent variables were selected according to preliminary assays: (i) number of extraction cycles (n), (ii) extraction time (min), and (iii) solid-to-solvent ratio (*w*/*v*). Each factor was evaluated at three levels, and a total of 15 experimental runs were performed, as shown in Table 4.

The experimental design allowed the assessment of both the individual and interactive effects of the selected variables on the response factors. The responses considered were total carotenoid yield (expressed as mg β-carotene per 100 g of dry weight, mg β-carotene/100 g DW) and antioxidant capacity, determined by DPPH and ORAC-FL assays.

Experimental data were fitted to a second-order polynomial model, and the adequacy of the model was evaluated by analysis of variance (ANOVA), including the determination of regression coefficients and statistical significance of the model terms. Statistical analysis and model generation were performed using Statgraphics 18 and Design-Expert 25 software.

### 3.5. Determination of Carotenoids

#### 3.5.1. Spectrophotometric Determination of Total Carotenoids

Total carotenoid content was determined spectrophotometrically at 450 nm using a UV-Vis spectrophotometer, Tecan Infinite 200 PRO multimode reader (Männedorf, Switzerland), following the method described by Biehler et al. [20]. Results were expressed as mg β-carotene per 100 g of dry weight (mg β-carotene/100 g DW) using the specific absorption coefficient of β-carotene (451 nm).

#### 3.5.2. Quantification of Individual Carotenoids by HPLC-DAD

Individual carotenoids were quantified by HPLC-DAD using external calibration curves constructed with commercially available standards of the target carotenoids (Carote Nature, Münsingen, Switzerland). Stock standard solutions were prepared according to the solubility of each compound: β-carotene was dissolved in hexane, while the remaining carotenoid standards were dissolved in diethyl ether. Working standard solutions were subsequently prepared by appropriate dilution in the mobile phase solvent mixture. Seven calibration levels were prepared for each carotenoid standard, covering a concentration range from 1 µg/mL to 1 mg/mL. Calibration curves were constructed by plotting peak areas versus concentration for each compound and were used for the quantification of individual carotenoids in papaya peel extracts.

Chromatographic separation and detection conditions were described in Section 2.3. Carotenoids were identified and quantified using commercially available authentic standards analyzed under the same chromatographic conditions. Peak identities were confirmed by comparison of both retention times and UV–Vis spectral characteristics obtained from the diode array detector (DAD) (JASCO Corporation, Tokyo, Japan). Quantification was performed using the corresponding external calibration curves. Attention was given to the identification and quantification of xanthophylls commonly associated with visual function, including lutein, zeaxanthin, violaxanthin, and β-cryptoxanthin. Results were expressed as mg of each carotenoid per 100 g of dry weight (mg/100 g DW).

### 3.6. Antioxidant Capacity Assays

#### 3.6.1. DPPH Radical Scavenging Activity

The antioxidant activity was measured by the DPPH (2,2-diphenyl-1-picrylhydrazyl) assay according to Brand-Williams et al. [21], with slight modifications. Briefly, an aliquot of the extract (Section 2.3) was mixed with a DPPH methanolic solution and incubated in the dark at room temperature for a fixed period to allow the reaction to reach a steady state. The decrease in absorbance was measured at 517 nm using a microplate reader (Tecan Infinite 200 PRO, Männedorf, Switzerland).

A calibration curve was constructed using Trolox as the standard, and the antioxidant activity was calculated by comparing the absorbance reduction in the samples with that of the Trolox solutions. Results were expressed as mmol Trolox equivalents per 100 g of dry weight (mmol TE/100 g DW).

#### 3.6.2. Oxygen Radical Absorbance Capacity (ORAC-FL)

The ORAC-FL assay was performed using fluorescein as the fluorescent probe and AAPH as the peroxyl radical generator, following Ou et al. [22]. Fluorescence decay was recorded with a microplate reader, the Tecan Infinite 200 PRO multimode reader, at excitation/emission wavelengths of 485/520 nm. The antioxidant capacity was calculated based on the Trolox standard curve and expressed as mmol TE/100 g DW.

The resulting fluorescence decay curves for samples, blanks, and Trolox standards were used to calculate the area under the curve (AUC) for each well. For each Trolox concentration, a standard calibration line (Net AUC versus Trolox concentration) was constructed. The antioxidant capacity of each extract was then obtained by interpolation on the Trolox curve using the sample’s Net AUC (after blank subtraction) and subsequently multiplied by the dilution factor to yield mmol TE/100 g DW [23].

#### 3.6.3. Ascorbic Acid Determination

The concentration of ascorbic acid (vitamin C) in the extracts was determined by redox titration using a standard volumetric method commonly applied for its quantification [24]. This analysis was performed to assess the potential contribution of vitamin C to the overall antioxidant capacity of the extracts. Results were expressed as mg of ascorbic acid per 100 g of dry weight (mg/100 g DW).

### 3.7. Statistical Analysis

All experiments were performed in triplicate, and results were expressed as mean ± standard deviation (SD). Data analysis was carried out using Statgraphics 18 Centurion software. One-way analysis of variance (ANOVA) was applied to evaluate significant differences among treatments, and Tukey’s post hoc test was used for multiple comparisons at a confidence level of *p* < 0.05.

For the optimization of extraction conditions, Response Surface Methodology (RSM) was applied using a Box–Behnken design. The adequacy of the models was assessed by ANOVA analysis, determination coefficient (R^2^), and the lack-of-fit test. Predicted values were compared with experimental data to assess the model’s reliability.

## 4. Conclusions

The present study positions papaya peel as a promising source of carotenoids within a broader framework of sustainable bioresource valorization. The sequential approach, from extraction optimization to chromatographic characterization, demonstrated that this agro-industrial by-product contains a nutritionally relevant carotenoid profile, particularly lutein and zeaxanthin, compounds that have been associated with visual health in previous studies.

The extraction results revealed that VAE provided significantly higher carotenoid recovery than UAE and MAE under the evaluated conditions. This finding is particularly relevant from a process development perspective, as it indicates that simple, low-energy, and scalable methodologies can efficiently recover carotenoids from papaya peel without requiring more complex assisted-extraction systems.

Chromatographic analysis further confirmed the predominance of xanthophyll carotenoids, with lutein identified as the major compound, followed by violaxanthin, zeaxanthin, β-cryptoxanthin, and β-carotene. The abundance of these carotenoids is especially relevant considering their recognized antioxidant properties and their association with visual and macular health. In contrast, the absence of lycopene suggests a cultivar- and tissue-dependent carotenoid distribution in papaya fruits.

The nutritional relevance of these findings is reinforced by the fact that lutein and zeaxanthin are among the principal carotenoids included in AREDS2-based nutritional formulations for age-related macular degeneration. Although the concentrations detected in papaya peel are substantially lower than those provided through clinical supplementation, the regular incorporation of carotenoid-rich ingredients derived from this by-product could contribute to dietary carotenoid intake.

Importantly, this work also supports circular economy strategies by transforming an underutilized agro-industrial residue into a potential source of high-value bioactive compounds. Therefore, papaya peel represents a promising candidate for developing sustainable functional ingredients with potential applications in food, nutraceutical, and health-oriented formulations.

## Figures and Tables

**Table 1:** Total carotenoid content of papaya peel extracts obtained under different experimental conditions.

Total Carotenoid Content of Papaya Peel		
Experiments	(mg β-carotene/100 g DW)	
	EV	PV
1	6.76 ± 0.46	7.18
2	8.02 ± 0.35	8.24
3	4.07 ± 0.25	3.84
4	2.12 ± 0.23	2.8
5	9.31 ± 0.60	9.77
6	5.89 ± 0.54	6.34
7	4.64 ± 0.25	3.96
8	8.93 ± 0.41	9.61
9	4.39 ± 0.33	4.58
10	3.46 ± 0.51	3.01
11	8.55 ± 0.59	7.18
12	9.16 ± 0.74	8.7
13	15.02 ± 0.66	10.82
14	6.23 ± 0.39	8.18
15	13.54 ± 0.71	12.86
R^2^	0.954	
Standard error of the estimate	1.2	
Durbin–Watson statistic	1.9 (*p* = 0.426)	

EV: experimental values. PV: predicted values obtained from the fitted second-order polynomial model. R^2^: coefficient of determination of the model. The standard error of estimate and the Durbin–Watson statistic are also reported to evaluate model accuracy and residual independence.

**Table 2:** Antioxidant activity of papaya peel extracts obtained under different experimental conditions defined by the Box–Behnken design, evaluated by DPPH and ORAC-FL assays.

Experiments	Antioxidant Activity of Papaya Peel			
	DPPH		ORAC-FL	
	(mmol TE/100 g DW)		(mmol TE/100 g DW)	
	EV	PV	EV	PV
1	337.9 ± 23.2	490.0	4.3 ± 0.6	5.2
2	727.8 ± 49.9	702.2	6.7 ± 0.9	6.1
3	322.8 ± 30.4	347.9	2.5 ± 0.2	3.2
4	228.3 ± 32.7	292.6	1.8 ± 0.2	2.3
5	599.0 ± 73.0	618.1	4.2 ± 0.5	5.0
6	519.1 ± 52.3	608.1	5.5 ± 0.6	6.6
7	421.2 ± 35.5	426.1	3.2 ± 0.6	3.0
8	626.9 ± 46.3	620.4	4.9 ± 0.3	5.1
9	369.6 ± 26.3	299.3	4.5 ± 0.2	4.3
10	311.2 ± 32.7	290.9	4.4 ± 0.4	3.5
11	579.2 ± 51.5	489.6	5.5 ± 0.6	5.2
12	671.5 ± 50.7	581.0	6.2 ± 0.6	5.1
13	752.26 ± 45.9	821.1	7.6 ± 0.5	7.8
14	553.4 ± 41.5	489.6	5.9 ± 0.7	5.2
15	906.4 ± 48.3	841.1	7.7 ± 0.8	7.3
R^2^	0.867		0.84	
Standard error of the estimate	30		0.6	
Durbin–Watson statistic	1.5 (*p* = 0.076)		1.5 (*p* = 0.117)	

EV: experimental values. PV: predicted values obtained from the fitted second-order polynomial model. R^2^: coefficient of determination of the model for each response. The standard error of estimate and the Durbin–Watson statistic are also reported to assess model accuracy and residual independence.

**Table 3:** Validation of optimized extraction conditions for carotenoid yield and antioxidant activity in papaya peel.

Response Parameter	Optimal Extraction Conditions ^a^	Random Extraction Conditions ^a^
	3 Cycles. 0.40 *w*/*v* Ratio. 1.25 min Vortex	2 Cycles. 0.25 *w*/*v* Ratio. 1.5 min Vortex
Carotenoid content (mg β-carotene/100 g DW)	13.54 ± 0.71	6.76 ± 0.46
Antioxidant activity		
DPPH (µmol TE/100 g DW)	906.4 ± 48.3	337.9 ± 23.2
ORAC-FL (mmol TE/100 g DW)	7.7 ± 0.8	4.3 ± 0.6

^a^ The extraction variables were extraction time, number of extraction cycles, and solid-to-solvent ratio (*w*/*v*).

**Table 4:** Experimental design: combinations of vortex time (min), number of extraction cycles (n), and solid-to-solvent ratio (*w*/*v*) used in the response surface methodology.

Experiments	n	*w*/*v*	t (min)
1	2	0.25	1.5
2	2	0.40	2.0
3	2	0.10	1.0
4	1	0.10	1.5
5	1	0.40	1.5
6	3	0.25	2.0
7	1	0.25	2.0
8	3	0.25	1.0
9	2	0.10	2.0
10	3	0.10	1.5
11	2	0.25	1.5
12	1	0.25	1.0
13	2	0.40	1.0
14	2	0.25	1.5
15	3	0.40	1.5

## Data Availability

The original contributions presented in the study are included in the article; further inquiries can be directed to the corresponding authors.

## Abbreviations

The following abbreviations are used in this manuscript:

RSM	Response surface methodology
ANOVA	Analysis of variance
AUC	Area under the curve
DAD	Diode array detector
DW	Dry weight
DPPH	2,2-diphenyl-1-picrylhydrazyl
FW	Fresh weight
MAE	Microwave-assisted extraction
ORAC	Oxygen Radical Absorbance Capacity
UAE	Ultrasound-assisted Extraction
